# Rescue of retinal ganglion cells in optic nerve injury using cell-selective AAV mediated delivery of *SIRT1*

**DOI:** 10.1038/s41434-021-00219-z

**Published:** 2021-02-15

**Authors:** Ahmara G. Ross, Devin S. McDougald, Reas S. Khan, Thu T. Duong, Kimberly E. Dine, Puya Aravand, Jean Bennett, Venkata Ramana Murthy Chavali, Kenneth S. Shindler

**Affiliations:** 1 University of Pennsylvania/Ophthalmology, Philadelphia, PA USA; 2 Center for Advanced Retinal and Ocular Therapeutics, F. M. Kirby Center for Molecular Ophthalmology, Perelman School of Medicine, University of Pennsylvania, Philadelphia, PA USA

**Keywords:** Neuroscience, Biological techniques

## Abstract

SIRT1 prevents retinal ganglion cell (RGC) loss in models of optic neuropathy following pharmacologic activation or genetic overexpression. The exact mechanism of loss is not known, prior evidence suggests this is through oxidative stress to either neighboring cells or RGC specifically. We investigated the neuroprotective potential of RGC-selective SIRT1 gene therapy in the optic nerve crush (ONC) model. We hypothesized that AAV-mediated overexpression of SIRT1 in RGCs reduces RGC loss, thereby preserving visual function. Cohorts of C57Bl/6J mice received intravitreal injection of experimental or control AAVs using either a ganglion cell promoter or a constitutive promoter and ONC was performed. Visual function was examined by optokinetic response (OKR) for 7 days following ONC. Retina and optic nerves were harvested to investigate RGC survival by immunolabeling. The AAV7m8-SNCG.SIRT1 vector showed 44% transduction efficiency for RGCs compared with 25% (*P* > 0.05) by AAV2-CAG.SIRT1, and AAV7m8-SNCG.SIRT1 drives expression selectively in RGCs in vivo. Animals modeling ONC demonstrated reduced visual acuity compared to controls. Intravitreal delivery of AAV7m8-SNCG.SIRT1 mediated significant preservation of the OKR and RGC survival compared to AAV7m8-SNCG.eGFP controls, an effect not seen with the AAV2 vector. RGC-selective expression of SIRT1 offers a targeted therapy for an animal model with significant ganglion cell loss. Over-expression of SIRT1 through AAV-mediated gene transduction suggests a RGC selective component of neuro-protection using the ONC model. This study expands our understanding of SIRT1 mediated neuroprotection in the context of compressive or traumatic optic neuropathy, making it a strong therapeutic candidate for testing in all optic neuropathies.

## Introduction

The irreversible effects of traumatic optic neuropathy (TON) can result from direct impulse or indirectly by shearing or stretch of the optic nerve [1]. Although this type of injury can occur in any age group, most TON occurs in young males under the age of 18, resulting in a range of visual outcomes from unaffected to substantial vision loss. The damage occurs at the level of axons, disrupting critical connections needed to maintain retinal ganglion cell (RGC) function. Mechanisms such as vascular and inflammatory mediators of RGC death have been implicated in perpetuating the process of RGC death, however the true pathophysiology is likely direct cell death [2]. Regardless, it is clear there is an unmet need for therapy aimed at delaying and potentially aborting the cell death associated with loss of vision.

Any primary insult to the visual tract or secondary deficits due to inflammation, demyelination, compression, or structural effects of the surrounding tissue can lead to RGC death and visual decline. Optic nerve crush (ONC) induces axonal degeneration, followed by a gradual death of RGCs, which results in irreversible vision loss [3], providing a fast, reproducible technique to observe RGC decline, as well as observe effects of novel neuroprotective therapeutic approaches [4].

A multitude of data has implicated a role for *SIRT1*-induced mitochondrial biogenesis in RGC survival in ONC, inflammatory, and viral-induced optic neuropathies [4–7]. Enhanced activity and overexpression of this gene have shown delayed RGC death resulting in visual decline in multiple optic nerve injuries. Recently, increased expression of *SIRT1* using AAV vectors with non-selective expression in retinal cells demonstrated a sustained and upward trend in neuro-protective capabilities [8] making this an excellent initial target gene for validating the use of this technology. This therapeutic intervention was not RGC specific.

The use of gene therapy as a delivery system to treat ophthalmic diseases continues to show promise as a powerful technique to introduce targeted changes into a cell with molecular surgical precision [9]. Ophthalmology represents an area where this technique has flourished due to the eye’s accessible location and the ability of progress to be monitored in vivo with minimal damage to tissues. One particular area of ophthalmology that would benefit from gene targeting is optic neuropathy [10]. The mitochondrial biogenesis and RGC survival induced by *SIRT1* in optic neuropathies makes it an excellent target gene for developing a gene therapy treatment.

In the present study, we compared the effects of *SIRT1* overexpression in experimental ONC via AAV gene transfer to RGCs. We developed and characterized AAV7m8 vectors that drive RGC-specific expression of human *SIRT1* in vitro and in the mouse retina. We examined the neuroprotective contribution of *SIRT1* gene augmentation in suppressing RGC death and vision loss in mice that have undergone ONC.

## Methods

### Animals

C57Bl/6J mice were obtained from the Jackson Laboratory and raised in a 12-h light/dark cycle. Animals were housed at the University of Pennsylvania in compliance with ARVO Statement for the Use of Animals in Ophthalmic and Vision Research as well as with institutional and federal regulations.

### Cell culture

The human iPSCs were generated from keratinocytes or blood cells via polycistronic lentiviral transduction (Human STEMCCA Cre-Excisable constitutive polycistronic [OKS/Myc] Lentivirus Reprogramming Kit, Millipore) by University of Pennsylvania iPSC Core facility and characterized with a hES/iPS cell pluripotency RT-PCR kit [11]. The induced pluripotent stem cell-derived retinal ganglion cells (iPSC-RGCs) were derived using a modified protocol involving small molecules to inhibit BMP, TGF-ß (SMAD) and Wnt signaling to differentiate RGCs from iPSCs [12]. The iPSC-RGCs cells with structural and functional features characteristic of native RGCs are used in our study.

We seeded iPSC-RGCs cells at a density of 350,000 cells and transduced with AAV2 vectors at a multiplicity of infection (MOI) of 100,000 vector genomes (vg) per cell. iPSC-RGCs were harvested 48 h post-transduction for immunocytochemistry analysis. Cells were rinsed with 1X PBS and fixed in 4% paraformaldehyde (PFA) for 15 min at room temperature. Afterwards, cells were blocked in 0.1% Triton X-100 and 1% bovine serum albumin (BSA) for 30 min at room temperature. Cells were incubated with primary antibody solution in 1% BSA and rabbit anti-FLAG antibody (CST #14793; 1:200), *SIRT1* antibody (sc-74465; 1:200), or BRN3A (EP1972Y, 1:2000) for 1 h at room temperature. Cells were washed with 1X PBS and incubated in secondary antibody solution containing 1% BSA and goat anti-rabbit AlexaFluor-594 antibodies (1:500) for 1 h at room temperature. Cells were removed from secondary incubation, washed in 1X PBS, and mounted with (Fluoromount-G; Southern Biotech; Birmingham, AL, USA) containing DAPI.

### AAV vector design and production

Human *SIRT1* (transcript variant 1) cDNA clones were obtained from Origene. Sequences were amplified with Q5 DNA polymerase (NEB) and cloned into an AAV expression plasmid using a commercial cloning kit (In-Fusion HD; Clontech Laboratories, Mountain View, CA, USA). Transgene expression was driven by either the CAG promoter derived from pDRIVE-CAG (InvivoGen, San Diego, CA, USA) [13] or the codon optimized SNCG (gamma-synuclein promoter) [13]. Both cDNA sequences contained a C-terminal 3xFLAG epitope tag that terminates into a bovine growth hormone polyadenylation sequence. AAV expression cassettes were flanked by the AAV2 inverted terminal repeats. A pro-viral plasmid driving expression of enhanced green fluorescent protein (eGFP) was used [8] and contains identical cis regulatory elements. AAV2-CAG.SIRT1, AAV2-CAG.eGFP, AAV7.m8-SNCG.SIRT1, AAV7m8-SNCG.eGFP vectors were generated using previously described methods and purified with CsCl gradient by the CAROT research vector core at the University of Pennsylvania [14]. The AAV7m8 capsid plasmid was a kind gift from Dr. John Flannery (UC-Berkeley).

### Intravitreal injections

4-week-old mice were anesthetized by isoflurane inhalation. A 33½ gauge needle was used to create a small incision at the limbus. Afterward, a 10-uL Hamilton syringe (701 RN; Hamilton Company, Reno, NV, USA) attached to a 33-gauge blunt-end needle was inserted into the vitreous cavity with the needle tip placed directly above the optic nerve head. We dispensed 2 uL of AAV preparation containing approximately 1 × 10^10^ vector genomes into each eye bilaterally. Vehicle treated eyes were injected with an equivalent volume of vector dilution buffer (0.001% Pluronic F68 in PBS). The two eyes of each mouse received different injections (vehicle, AAV2-CAG.SIRT1, AAV7m8-SNCG.SIRT1, AAV2-CAG.eGFP, or AAV7m8-ANCG.eGFP) allowing each eye to serve as an independent experimental end point.

### Optic nerve crush

Optic nerve crush (ONC) was performed on 12-week-old C57Bl/6J wild-type mice as in our prior studies [4]. Mice were anesthetized systemically with xylazine and ketamine and topically with 0.5% proparacaine eye drops. Under a dissecting microscope, the conjunctiva was lifted with fine forceps and cut using scissors, exposing the sclera. Forceps were used to manipulate and retract orbital fat and muscles, allowing for exposure of the optic nerve. Injury to the optic nerve was induced using curved fine-tip forceps to induce a focal crush injury to the optic nerve ~1 to 2 mm behind the globe. Maximal pressure was used to close the forceps for 1 s. Use of fine-tip forceps facilitated avoidance of orbital vessels to avoid ocular ischemia. Bleeding during the procedure was categorized as none, minimal, moderate, and large. Mice were excluded for more than moderate bleeding. In each experiment, the ONC was performed in one eye only, allowing the contralateral uninjured eye to serve as a control. The surgeon performing ONC was masked to eyes that received the AAV constructs.

### Optokinetic response recordings (OKRs)

Visual function was assessed by measuring the OKR using commercial software and apparatus (OptoMetry; CerebralMechanics, Inc., Medicine Hat, AB, Canada) as previously described [15]. OKR was determined as the highest spatial frequency where mice track a 100% contrast grating that is projected at different spatial frequencies. Measurements were performed by an investigator masked to the experimental treatments. Each datapoint represents ten animals in experiments performed in triplicate.

### Retinal histology and RGC quantification

Eyes were harvested and placed in 4% PFA overnight at 48 °C. Eyes were washed in PBS followed by dissection of retinal cups. Tissues were permeabilized and blocked in 2% Triton X-100, 10% normal bovine serum, and PBS and then incubated with goat anti-Brn3a antibody (Novus Biologics) diluted 1:100 at 48 °C. Retinal cups were washed and then incubated in secondary antibody solution containing 2% Triton X-100, 10% normal bovine serum, and donkey anti-goat AlexaFluor 594 antibody (1:500 dilution). After washing, samples were prepared as flat mounts and mounted onto glass slides with an aqueous mounting medium (SouthernBiotech) containing 4′,6-diamidino-2-phenylindole (DAPI). RGCs were quantified as previously described [5, 6, 16, 17]. Briefly, retinal micrographs were recorded at ×40 magnification in 12 standard fields (from the center of the retina in each quadrant). Total RGC counts from the 12 fields per retinal sample covering a total area of 0.45 mm^2^/retina were recorded by an investigator masked to the experimental conditions using Photometrics cell counting software. Retinal cross-sections were incubated in blocking buffer containing PBS, 2% Triton X-100, and 10% normal donkey serum for 1 h at room temperature. Next, sections were incubated in primary antibody solution containing the previously described components and a rabbit anti-FLAG antibody (CST #14793) at 1:100 dilutions overnight in a humidified chamber at room temperature. Sections were washed in PBS three times and incubated in secondary antibody solution containing donkey anti-rabbit AlexaFluor 488 antibody diluted at 1:200 for 2 h at room temperature. Slides were then washed in PBS three times and mounted with aqueous mounting medium (SouthernBiotech) containing DAPI. Retinal whole mount photography and counting of RGCs was performed by a masked investigator. Each experiment represents ten animals per group with experiments performed in triplicate.

### Axon analysis

Neurofilament staining was performed and quantified in longitudinal paraffin embedded sections. Briefly, optic nerves were isolated, processed and embedded in paraffin. 5 μm longitudinal paraffin sections were deparaffinized, rehydrated, and nonspecific binding was reduced using Blocking reagent (Vector Laboratories, Burlingame, CA, USA). Specimens were then incubated in rabbit anti-neurofilament antibody 1:500 (Abcam, Cambridge, MA, USA) at 4 °C overnight. Sections were washed three times with PBS, then incubated with anti-rabbit secondary antibody (Vectastain Elite ABC Rabbit kit) for 30 min at 37 °C. Avidin/Biotin Complex detection was performed by incubating with Vectastain Elite ABC reagent at 37 °C for 30 min and DAB (diaminobenzidine, Vector labs) substrate for 3 min at RT followed by washing in running water for 5 min. Dehydrated slides were mounted using Refrax mounting medium. Photographs of three fields/nerve (one each at the distal, central, and proximal regions of the longitudinal optic nerve section) at ×40 magnification were taken by a masked investigator. Neurofilament staining optical density was quantified by using ImageJ software (http://nih.gov).

Bisected optic nerves were incubated in 2% osmium tetroxide and dehydrated in graded ethanol immersions. The nerves were then embedded in epoxy resin Embed 812 (Electron Microscopy Sciences, Hatfield, PA), 0.75 μm thick cross sections were generated from a section of the nerve 1.5 mm posterior to the globe and stained with 1% toluidine blue. Each optic nerve cross section was analyzed at five standardized photomicrographs (75 × 75 μm) obtained at ×100 magnification: one in the center and in four quadrants. Axon counts were obtained by a masked operator using the AxonJ ImageAnalysis algorithm plugin for ImageJ (http://imagej.nih.gov/ij/plugins/axonj/) [18]. Mean ± standard error of the mean (SEM).

### Statistics

All data are represented as mean ± SEM. Each experiment was repeated 3 times for statistical analysis. Differences between treatment groups with respect to OKR responses, RGC quantification, and optic nerve histopathology were compared using a 1-way ANOVA followed by Tukey’s honest significant difference test using statistical software (GraphPad Prism 5.0; GraphPad Software, Inc., La Jolla, CA, USA). Differences were considered statistically significant at *P* < 0.05. Data meet the assumption of normal distribution of tests with variances between GFP and SIRT1 groups. No randomization was performed; however investigators were masked to disease and therapeutic intervention to quantify endpoints.

## Results

### Design and characterization of AAV7m8 vectors

**Fig. 1:**
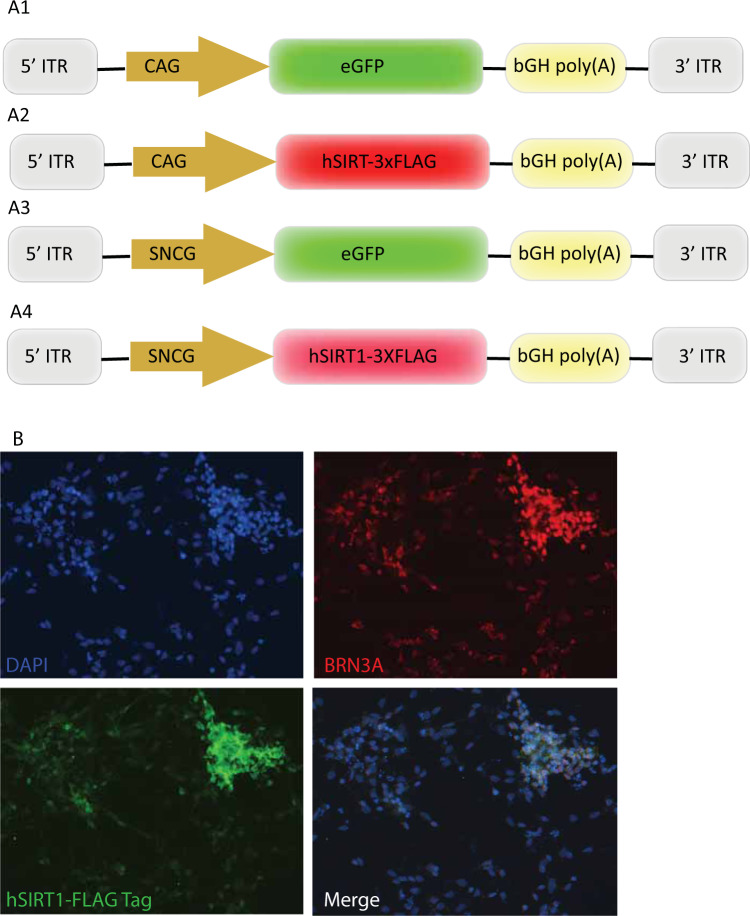
Design and in vitro characterization of AAV2-CAG and AAV7m8 SNCG vectors. **A** Outline of proviral expression cassettes used in the study. A1 and A2 illustrates comparative vector using AAV2 and CAG promoter with the cDNAs encoding eGFP or SIRT1 respectively. A3 and A4 Illustrates plasmid using the ganglion cell specific promoter, SNCG, driving cDNAs eGFP or SIRT1 respectively. **B** Fluorescent micrographs of human SIRT1 protein expression in iPS-RGCs transduced with AAV7m8.SNCG.hSIRT1-3XFLAG. Blue-DAPI nuclear stain, Red-BRN3A nuclear stain, green- 3XFlag Tag epitope showing cytoplasmic and nuclear expression from the vector and blue/red/green merged images.

We generated AAV2 and AAV7m8 vectors expressing eGFP and a target gene human *SIRT1* driven by the CAG promoter or a RGC-selective gamma synuclein promotor [13] (Fig. 1A1–4). Using induced pluripotent stem cell derived RGCs (IPS-RGCs) developed in a manner previously described [19] and collected from a healthy visually unaffected male, age 32, vector expression was examined in vitro using immunofluorescent labeling on iPSC-RGCs (Fig. 1B1–4). This revealed robust cell number (Fig B1) in cultures of viable iPS-RGCs (Fig. B2), with notable levels of gene expression with cytoplasmic and nuclear distribution of the tagged protein (Fig. B3).

**Fig. 2:**
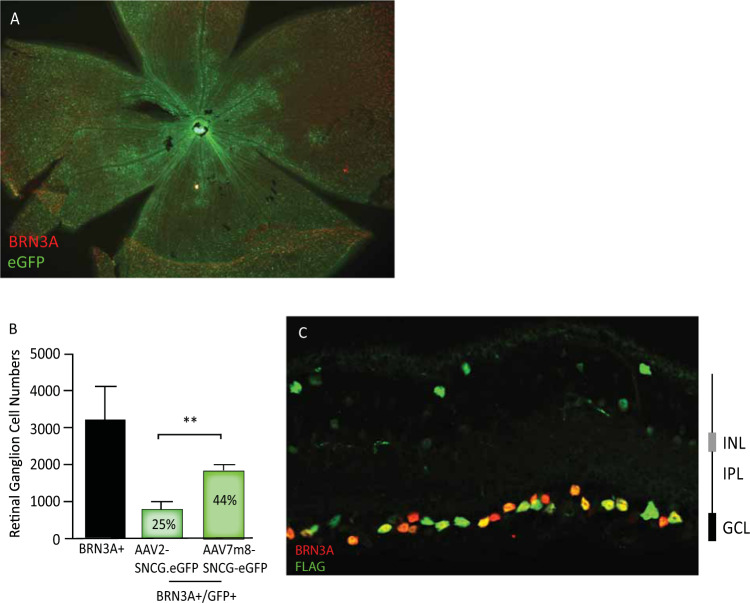
AAV7m8 transduction profile and RGC transduction efficiency following intravitreal delivery. **A** Representative micrograph of retinal flat mount following intravitreal injection of AAV7m8-SNCG.eGFP. RGCs are labeled with BRN3A (red). (inlet) Representative retinal flat mount used for calculating RGC transduction efficiency with AAV7m8. **B** Quantification of RGC transduction (*n* = 10, experiments performed in triplicate; retinal whole mounts) comparing AAV2-CAG.SIRT1 vector. **C** Representative cross-section of mouse retina following intravitreal injection of AAV2-CAG.SIRT1. RGCs are labeled with Brn3a (red) and localized to the ganglion cell layer(GCL). Cells expressing the SIRT1 transgene are labeled green and also largely localized to the GCL.Data represented as mean ± SEM.

Next, we compared the retinal transduction profile of AAV2-CAG.eGFP to AAV7m8-SNCG.eGFP following intravitreal delivery with a vector expressing enhanced green fluorescent protein in a cohort of wild-type mice as previously described [20]. This vector was compared to a previously described vector developed with a ubiquitous CAG promoter expressing the same human and codon optimized target gene *SIRT1* but packaged in an AAV2 capsid. The AAV2.7m8-eGFP vector displayed a higher transduction efficiency of the ganglion cell layer than previously published [8]. Compared with the 25% RGC transduction displayed by the AAV2-CAG.eGFP vector, AAV7m8-SNCG.eGFP achieved ~44% RGC transduction by quantifying the number of eGFP positive RGCs labeled with BRN3A antibody in retinal whole mounts (Fig. 2A1, A2). AAV7m8 vectors driving expression of *SIRT1* were injected in to the right and left eyes, respectively, all wild-type mice displayed similar transduction profiles in vivo (Fig. B1 and B2).

### Constitutively expressed *SIRT1* gene transfer using AAV2 vector does not rescue RGC protection after ONC

**Fig. 3:**
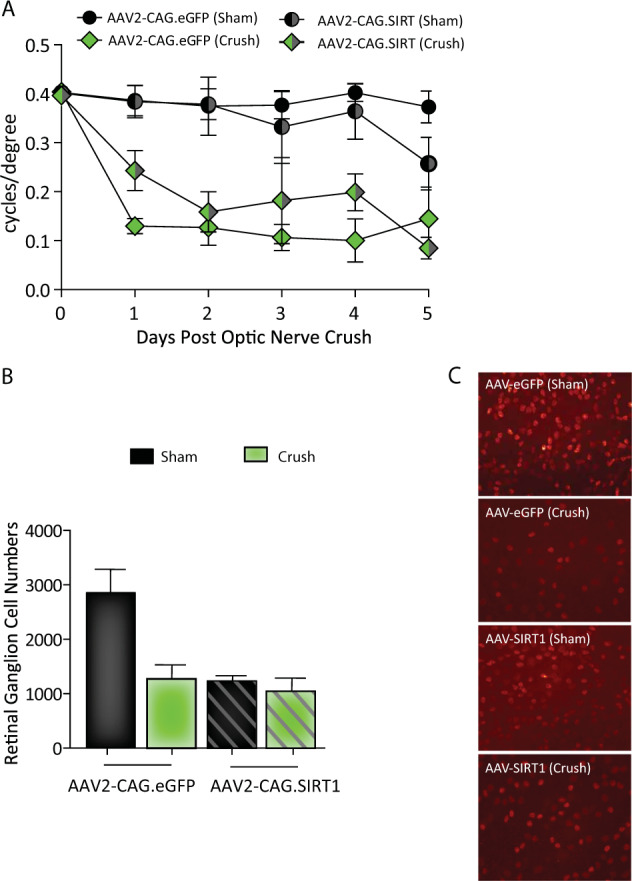
Effect of AAV2 gene transfer on visual acuity and RGC in ONC. OKR recordings demonstrate significantly decreased visual acuity in eyes of ONC mice treated with AAV2-CAG.eGFP (*n* = 10; experiments performed in tripicate). Treatment with AAV2- Mice treated with AAV2-CAG.SIRT1 (*n* = 12; experiments performed in tripicate) show no significant effect on **A** visual acuity or **B** RGC survival by day 7. **C** Representative RGC counts by nuclear BRN3A staining. Data represented as mean ± SEM. *P < 0.05, **P < 0.01 by 1-way ANOVA with Tukey’s HSD post-test.

C57Bl6/J mice received intravitreal injections of experimental or control AAV2 vectors at postnatal week 4 followed by optic nerve crush (ONC) induction at postnatal week 12 (Fig. 3A). Following ONC [4, 21] we measured visual function by OKR daily. Control treated animals treated post-intravitreal injections of AAV2-CAG.eGFP exhibit normal OKR scores (Fig. 3A) and retained RGCs (Fig. 3B, C) throughout the experimental timeline (Fig. 3A) which suggests no adverse effects associated with delivery or overexpression of the control transgene. Similarly, animals injected with AAV2-CAG.SIRT1 displayed strong responses prior to induction. Following optic nerve trauma, ONC animals exhibit a decline in OKR scores beginning at day 1 post trauma, which is sustained through the experimental timeline. AAV2-CAG.SIRT1 transfection demonstrated no significant protection of visual acuity throughout the experimental timeline (Fig. 3A).

Permanent visual decline observed in ONC uniformly results in loss of RGC numbers [4, 21]. Retinas from each treatment group were stained with antibodies directed against Brn3a to determine whether *SIRT1* augmentation conferred a protective advantage upon RGCs during ONC (Fig. 3C). Intravitreal injection of AAV2 was well tolerated as indicated by comparative total RGC counts in sham-induced animals treated with eGFP. In mice traumatized with ONC, RGC numbers were significantly reduced in all treatment groups compared to sham-induced controls. This demonstrated no significant effect of *SIRT1* overexpression using a ubiquitous CAG driven promoter on RGC survival that transfects only 24% of RGCs (Fig. 2).

### Ganglion cell-selective expression of *SIRT1* mediated by AAV7m8 vector with the SNCG delays loss of visual function and protects against RGC loss after ONC

**Fig. 4:**
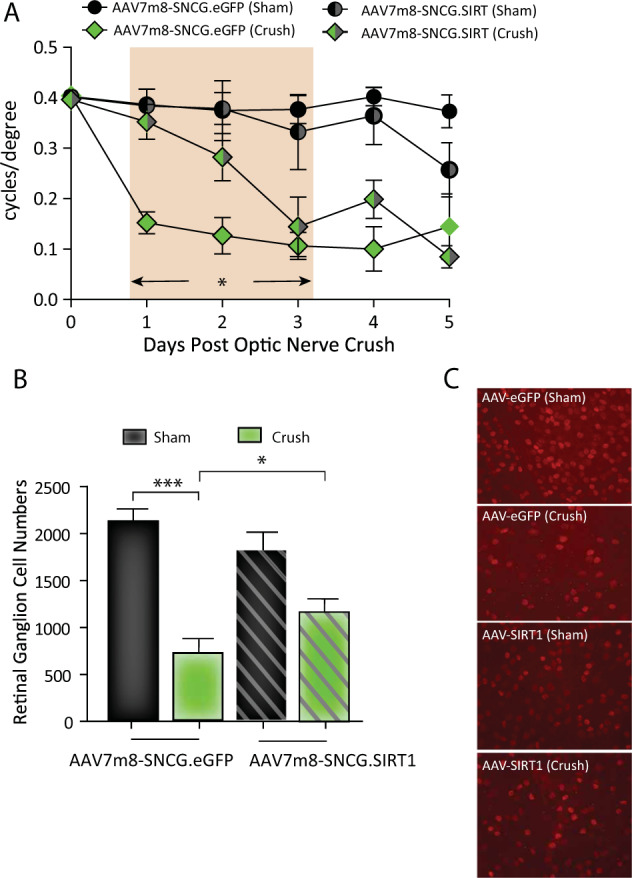
Effect of AAV7m8 gene transfer on visual acuity and RGC in ONC. OKR recordings demonstrate significantly decreased visual acuity in eyes of ONC mice treated with AAV2-eGFP (*n* = 10; experiments performed in triplicate) compared with Sham injured mice (*n* = 10; experiments performed in tripicate ***p = 0.002). **A** Treatment with AAV7m8-SIRT1 (*n* = 15; experiments performed in tripicate) showed a significant delay in loss of visual function in ONC (*n* = 10; experiments performed in tripicate) compared with Sham injured mice (*n* = 10; experiments performed in tripicate) (*p = 0.03) on visual acuity seen in peach area outlined in the graph**. B** RGC flat mount counts demonstrate significantly decreased numbers in eyes of ONC mice treated with AAV2-eGFP (*n* = 10; experiments performed in tripicate) compared with Sham injured mice (*n* = 10; experiments performed in triplicate; ***p = 0.001). Treatment with AAV7m8-SIRT1 (*n* = 10; experiments performed in tripicate) showed a significant increase in retinal ganglion cell counted per flat month function in ONC (*n* = 7) compared with control ONC mice (*n* = 10; experiments performed in tripicate) (*p = 0.03). **C** Representative RGC counts by nuclear BRN3A staining Data represented as mean ± SEM. *P < 0.05, **P < 0.01 by 1-way ANOVA with Tukey’s HSD post-test.

C57Bl6/J mice received intravitreal injections of AAV7m8 experimental and control vectors at postnatal week 4 followed by ONC at postnatal week 12. Sham Intravitreal injections of AAV2.7m8-eGFP did not interfere with OKR scores prior to ONC (Fig. 4A). Similarly, animals injected with AAV2-SNCG.SIRT1 displayed strong responses prior to induction. Following optic nerve trauma, untreated or AAV7m8-SNCG.eGFP-treated ONC animals exhibit a decline in OKR scores beginning at day 1 post trauma, which is sustained through the experimental timeline. However, eyes treated with AAV7m8-SIRT1 demonstrate a delay in reduction of functional responses by day 2 (Fig. 4A), (AAV2-CAG.SIRT1 = 0.282 ± 0.015; AAV2-CAG.eGFP = 0.127 ± 0.028; *P* = 0.002). This effect was not sustained but was able to delay vision loss for 3 days.

Similar to experiments performed using AAV2-CAG mediated constitutive overexpression of *SIRT1*, retinas of each treatment group were stained with antibodies directed against Brn3a to determine whether *SIRT1* augmentation conferred a protective advantage upon RGCs during ONC (Fig. 4B). Intravitreal injection of AAV7m8-SNCG was well tolerated as indicated by comparative total RGC counts in control AAV2.eGFP-injected animals. Treatment with AAV7m8-SNCG.SIRT1 resulted in a statistically significant increase in RGC survival compared to control eyes treated with AAV7m8-SNCG.eGFP (Fig. 4B, C) by day 6 after ONC (AAV7m8-SNCG.eGFP = 711.6 ± 513.52; AAV7m8-SNCG.SIRT1 = 1159.14 ± 400.1; *P* = 0.0156).

## Discussion

Adeno-associated virus (AAV) vectors have become the standard for achieving stable gene transfer with a safe clinical profile, especially when targeted to neurons, when taking into account numerous factors including dose, capsid, cassette, and manufacturing process. AAV2-vectors encoding *RPE65* demonstrated a robust safety profile following subretinal delivery in human clinical trials for Leber congenital amaurosis type 2 [22, 23]. Our study compared the neuroprotective effects of RGC specific to a non-cell specific gene transfer in an experimental mouse model of ONC. Our results suggest that *SIRT1* driven by a RGC specific promoter delayed loss of visual acuity and enhanced RGC survival in ONC. Interestingly, even with notable loss of visual function (Fig. 3A) there are still significant RGCs present (Fig. 3B, C) suggesting that these cell bodies, although present, might be too damaged to convey visual signals. This finding begs further investigation into whether the constructs can facilitate regeneration of optic nerve axons over time. While using different transgene cassettes, both this change, as well as the cell selectivity resulted in a greater transduction efficiency as well as a more impressive therapeutic effect (Fig. 4). Results suggest that *SIRT1* overexpression specifically in RGCs plays a significant role in delaying loss of RGC function and reducing RGC death from optic nerve injury. While we recognize that the AAV2 construct has a different transduction profile than AAV7m8 using the same transgene, the RGC layer, after intravitreal inject of the construct should receive the highest concentration of the virus. We previously showed that this AAV2 construct is transduced in multiple retinal layers [8] at low levels, but even with that current results with the AAV7m8 construct strongly suggest that it is the level expression of SIRT1 in RGCs that is is critical and sufficient to promote their survival. These effects are similar to neuroprotective effects promoted by pharmacologic and small molecular modulators of the SIRT1 pathway, as seen in mice treated with resveratrol and ST266 during experimental optic neuritis [21, 24]. Together, these results suggest that modulating *SIRT1* can promote RGC survival in multiple forms of optic neuropathy.

Under conditions of oxidative stress, *SIRT1* is translocated to the nucleus and modulates activity of protein targets primarily involved in oxidative phosphorylation and mitochondrial biogenesis [25, 26]. This function is induced by activating PGC1-α, a transcription regulator of mitochondrial function and antioxidant metabolism [27]. In addition to these known functions, active *SIRT1* deacetylates and inhibits the transcription factor, p53, thereby downregulating apoptotic gene expression and thus improving cell viability [28]. Previous studies further demonstrate that preservation of RGCs through SIRT1 mechanisms involves reduction of oxidative stress. Studies with numerous mechanisms of cell stress to RGCs, including serum starvation, doxorubicin toxicity, and H_2_O_2_ exposure induced significant cell loss through increase of mitochondrial derived reactive oxygen species by proposed mechanisms involving promotion of mitochondrial biogenesis and function leading to increased superoxide dismutase activity [29]. With the combination of these mechanisms, *SIRT1* could be important for promoting neuroprotection by activation of molecular pathways to inhibit cell damage from many different mechanisms of RGC damage.

With ONC, we observed a statistically significant visual decline at day 1 post trauma with all AAV treated animals subjected to optic nerve insult, and this effect was significantly delayed in AAV7m8-SNCG.SIRT1 treated animals. Previously, Zuo et al. demonstrated a similar delay in loss of visual function after ONC in mice with constitutive overexpression of *SIRT1* in all cell types using genetically modified mice. It was unclear from that prior study whether SIRT1 expression was necessary in multiple retinal cell types in order to promote RGC survival, as opposed to overexpression of SIRT1 mainly restricted RGCs. The similar effects on RGC survival found in the current study suggest a key role of SIRT1 signaling specifically within RGCs in SIRT1-mediated neuroprotection.

With a body of literature demonstrating that treatment with small molecular activators of the *SIRT1* pathway, such as resveratrol, preserves visual function and RGC survival in mouse models of optic neuritis and optic nerve trauma [4–7, 17, 21, 24], the current results suggest that the AAV2.7m8-SIRT1 vector might have utility in multiple optic neuropathies. Future examination of potential neuroprotection in more chronic injury to the optic nerve, such as glaucoma, or acute injury, such as ischemic optic neuropathy, may be warranted. Our data demonstrate that with cell specificity using the SNGC promoter, protection of visual function and RGCs is better achieved, suggesting the importance of cell specificity in the design of potential neuroprotective gene therapies for optic neuropathies.

## Supplementary information

Figure Legend

Supplemental Figure 1
