# Supplementary material for: Rescue of retinal ganglion cells in optic nerve injury using cell-selective AAV mediated delivery of SIRT1
Source: Gene Ther. 2021 Feb 15;28(5):256–64. doi: 10.1038/s41434-021-00219-z (PMC8149296; doi:10.1038/s41434-021-00219-z)
Supplement: Supplementary file 1 — Figure Legend [file 41434_2021_219_MOESM1_ESM.docx]

**Supplemental Figure.** Effect of AAV7m8 gene transfer on axon status. **(A)** Neurofilament staining was used to evaluate axonal loss in sections of optic nerves isolated at day 7 post crush. The optical density of neurofilament staining, calculated by a masked investigator using the average of three equal-sized fields from each optic nerve, showed no significant decrease in optic nerves (N = 3 nerves) from mice in four groups, with ONC (AAV7m8.eGFP and AAV7m8.SIRT1) compared to optic nerves (N = 3 nerves) from control mice (AAV7m8.eGFP and AAV7m8.SIRT1). **(B)** A series of photographs of axon staining in three equal-sized fields from each optic nerve (one each at the distal, central, and proximal regions of the longitudinal optic nerve section) shows the normal degree of variability of neurofilament staining in optic nerves of ONC and control mice. **(C)** One central and 4 peripheral 75 × 75 μm images were sampled for each cross section **(D)** Bar graph showing axonal counts performed on higher magnification images the sham and control mice treated with AAV7m8.eGFP and AAV7m8SIRT. The AxonJ Image Analysis Algorithm plugin for ImageJ was used to count the number of axons.
